# METTL3-mediated m6A modification of circRNF220 modulates miR-330-5p/survivin axis to promote osteosarcoma progression

**DOI:** 10.1007/s00432-023-05455-x

**Published:** 2023-10-14

**Authors:** Feng Liu, Wen Li, Zhihui Jin, Jia Ye

**Affiliations:** Department of Orthopedics, Renmin Hospital of Wuhan University, Wuhan, 430060 China

**Keywords:** **C**ircRNF220, miR-330-5p, Survivin, Progression, Osteosarcoma

## Abstract

**Background:**

Circular RNAs (circRNAs) play a crucial role in regulating various physiological processes. However, the precise regulatory mechanisms of circRNF220s in osteosarcoma (OS) are not well understood.

**Methods:**

The abundances of circRNF220, miR-330-5p, and survivin were determined using qRT-PCR. To assess the m6A accumulation in circRNF220, a methylated RNA immunoprecipitation (Me-RIP) assay was conducted. Cellular multiplication, motility, and invasion were examined using the cell Counting Kit-8 (CCK-8), EdU, colony formation, Transwell, and wound-healing assays. The binding relationships were measured through RNA immunoprecipitation (RIP) and luciferase reporter assays. In vivo functionality was assessed using xenograft models.

**Results:**

CircRNF220 was identified as being overexpressed in both OS cells and tissues. In vitro experiments demonstrated that silencing circRNF220 impeded the proliferation, invasion, and motility of OS cells. Similarly, in vivo studies confirmed that downregulating circRNF220 inhibited the growth of OS. Further mechanistic investigations unveiled that METTL3-modulated circRNF220 regulated the progression of OS by upregulating survivin expression through acting as a sponge for miR-330-5p.

**Conclusion:**

The modulation of METTL3-regulated circRNF220 has been found to promote the progression of OS by modulating the miR-330-5p/survivin axis. This novel finding suggests a potentially unique approach to managing OS.

**Supplementary Information:**

The online version contains supplementary material available at 10.1007/s00432-023-05455-x.

## Introduction

Osteosarcoma (OS) is the most prevalent primary osseous malignancy, accounting for 10% of pediatric and juvenile solid tumors (Zhao et al. 2021). OS is a highly aggressive condition known for its ability to metastasize and infiltrate surrounding tissues. The mainstays of treatment for OS are intensive chemotherapy and surgery. The overall 5-year survival rate for OS ranges from 70 to 80% (Wang et al. 2020a; Hu et al. 2022). However, for individuals who present with lung metastases, the 5-year survival rate can be as low as 5–10% (Anderson 2016; Fan et al. 2022). Despite major breakthroughs in the molecular targeting of OS in recent decades, little progress in boosting survival has been accomplished in the last decade(Otoukesh et al. 2018). Hence, it is crucial to further investigate the regulatory mechanisms of OS and develop innovative therapeutic techniques to effectively address this issue.

Circular RNA (circRNA) is a unique RNA molecule that undergoes end-to-end splicing, lacking traditional 3′ or 5′ endings (Wu et al. 2020; Kristensen et al. 2019). Due to the absence of free ends, circRNA exhibits resistance to cleavage by exonucleases, resulting in its stable expression in the cytoplasm (Lu et al. 2019). Recent reports suggest that circRNA can function as a competitive endogenous RNA in tumors, counteracting the inhibitory effect of miRNA on target protein mRNAs. This can have an impact on gene-mediated cellular signaling and ultimately regulate the progression of different types of malignant tumors.

Initial studies have shown that the abnormal expression of specific circRNAs in OS can affect cell proliferation, invasion, and multidrug resistance (Wang et al. 2020b, 2021; Enuka et al. 2016). For example, circ_0001721 plays a role in promoting the malignant behavior of OS by targeting the miR-372–3p/MAPK7 pathway (Gao et al. 2020). Moreover, given the high tissue level in OS, circ_0003074 has the potential to serve as a diagnostic and therapeutic biomarker for OS (Lei and Xiang 2020). CircRNF220, also known as hsa_circ_0000066, has been identified to promote tumors in different types of cancers (Zhang et al. 2022a; Liu et al. 2021). In acute myeloid leukemia (AML) relapse, circRNF220 acts as a pathogenic factor by sponging miR-30a and upregulating MYSM1 (Liu et al. 2021). However, the specific regulatory mechanisms and functional role of circRNF220 in OS are still unknown.

The N6-methyladenosine (m6A) modification is a common epigenetic RNA modification that plays a regulatory role in various RNA functions and is associated with disease progression (Timoteo et al. 2020). The involvement of m6A methyltransferases (writer enzymes), demethylases (erasers), and m6A-binding proteins (readers) facilitates the modification process (He et al. 2019). It has been observed that both m6A and circRNAs are associated with human cancer (Wang et al. 2020c). For example, circ-CTNNB1 promotes the progression of OS by undergoing m6A modification through its interaction with RBM15 (Yang et al. 2023). However, the roles of m6A-modified circRNF220 in OS remain unknown.

The GEO dataset GSE140256 was analyzed in this study, revealing elevated levels of circRNF220 in OS tissues. Furthermore, we demonstrated that circRNF220 can enhance the progression of OS towards malignant phenotypes by activating the miR-330-5p/surviving pathway in an m6A‑dependent manner.

## Materials and methods

### Clinical samples

We obtained 32 pairs of OS and healthy para-carcinoma tissue samples from Renmin Hospital of Wuhan University. The aforementioned hospital’s Ethics Committee approved and reviewed investigations involving human samples and animals. All the patients signed an informed consent form.

### Cell culture and transfection

OS cell lines (SaOS-2, HOS) and healthy osteoblasts (hFOB1.19) were cultured in 10% fetal bovine serum (FBS)-containing Dulbecco’s modified eagle’s media (DMEM). The circRNF220 shRNA, METTL3 shRNA and inhibitor of miR-330-5p were purchased from Servicebio (Wuhan, China). The pcDNA3.1-survivin plasmid was synthesized by Genepharma (Shanghai, China). Lipofectamine™ 3000 (Invitrogen, Shanghai, China) was used as the transfection vehicle, and the transfection efficiency was determined by qRT-PCR.

### qRT-PCR

Total RNA extraction was carried out in accordance with the TRIzol (Invitrogen) protocol. PrimeScriptTM RT reagent Kit was used to synthesize cDNA, followed by qRT-PCR using TB Green Premix Ex TaqTM. We normalized the circRNA and mRNA levels against GAPDH, whereas the miRNA levels were normalized against U6. Supplementary Table 1 summarizes the primer information.

### Stability analysis

Briefly, 2 mg/mL of actinomycin D and 3 U/µL ribonuclease R (RNase R) were used to analyze the stability of circRNF220. After the given duration of culture, the circRNF220 and RNF220 mRNA expressions were assessed by qRT-PCR.

### Luciferase reporter assay

CircRNF220’s 3′-UTR and survivin having miR-330-5p’s possible binding site were inserted into the pGL6-miR vector (Genepharma, Shanghai, China). Followed by co-transfection of 293T cells with miR-330-5p mimic and reporter vectors. The luciferase activity was determined by qRT-PCR.

### RNA immunoprecipitation (RIP)

The Magna RIP kit was used to assess the enrichment of circRNF220. The lysate was incubated in RIP buffer containing anti-Ago2 or anti-IgG antibody-bound magnetic beads. Following RNA purification, qRT-PCR was utilized to determine the levels of miR-330-5p and circRNF220.

### Fluorescence in situ hybridization (FISH) assay

MiR-330-5p and circRNF220 probes were synthesized and acquired from GenePharma (Shanghai, China). The OS cells were hybridized with the above probes overnight at 37 °C per the manufacturer’s instructions. The images were then captured with immunofluorescence microscopy.

### Cell proliferation

Following transfection, 96-well microplates were used to inoculate the OS cells, which were then treated with the CCK-8 solution (10 µl). The optical density (OD) at 450 nm was determined by a microplate reader. In addition, the cellular proliferative potential was evaluated using an EdU assay (Beyotime, China). Following a 30 min incubation of OS cells in EdU solution (100 µL), nuclei were stained with 300 µL DAPI solution for 5 min. Finally, cell images were taken by fluorescence microscopy (Zeiss, Germany).

### Wound healing assay

After transfection, the transfected OS cells were inoculated into each well of a 6-well microplate. Then using a 10 µL pipette tip, a linear wound is created in each well. The cell’s migratory potential was microscopically evaluated at different time points.

### Colony formation assay

The six-well microplates were inoculated with the post-transfected OS cells. After 2 weeks of incubation, colonies were fixed using methanol and stained with 0.1% crystal violet. Under microscopic observation, we enumerated the colonies and manually counted those consisting of at least 50 cells.

### Transwell assay

In brief, the upper chamber of a six-well Transwell plate was seeded with 5 × 10^5^ cells in serum-free medium, while a medium containing 20% FBS was dispensed into the bottom chamber. After that, the cells were immobilized in both chambers with paraformaldehyde (4%) and stained with crystal violet (0.1%). Finally, cells were quantified using a microscope.

### Western blot

RIPA buffer was utilized to extract the total cellular proteins. Equal amounts of proteins from different samples were run on an SDS-PAGE gel, followed by transferring them onto the polyvinylidene difluoride (PVDF) membrane. The PVDF membrane was then incubated overnight with a blocking buffer followed by incubation at 4 °C with 1:1000 dilution of primary antibodies, anti-GAPDH, and anti-survivin (Proteintech). Finally, after secondary antibody incubation, Image Lab (Bio-Rad) was used to photograph images.

### Methylated RNA immunoprecipitation (Me-RIP) assay

Total RNA was extracted using TRIzol reagent, and anti-m6A antibody or immunoglobulin (IgG) bound protein A/G magnetic beads were used. The total RNA was washed with elution buffer to isolate m6A-modified RNA. Subsequently, qRT-PCR analysis was conducted to assess the m6A accumulation in circRNF220.

### Xenograft tumor formation

Murine experimentation was approved by the Renmin Hospital ethics committee of Wuhan University. Briefly, mice were injected with 3 × 106 HOS cells having sh-NC or sh-circRNF220. Five weeks later, the mice were killed and their tumor weights were recorded. Following that the tumors were subjected to immunohistochemistry and immunofluorescence staining.

### Bioinformatics analysis

The GEO database was used to search for circRNAs that expressed differentially in OS versus healthy tissues. Starbase 2.0 was used to predict potential binding sites for miR-330–5p, circRNF220, and survivin.

#### Statistical analysis

Data were analyzed statistically via SPSS Ver. 22.0. Correlations among miR-330-5p, circRNF220, and survivin were evaluated through Pearson’s correlation analysis. Inter-group comparisons were made by Student’s t-test (two-tailed) or one-way ANOVA. Differences were regarded as significant when *P* < 0.05.

## Results

### CircRNF220 expression increases in OS cells and tissues

**Fig. 1:**
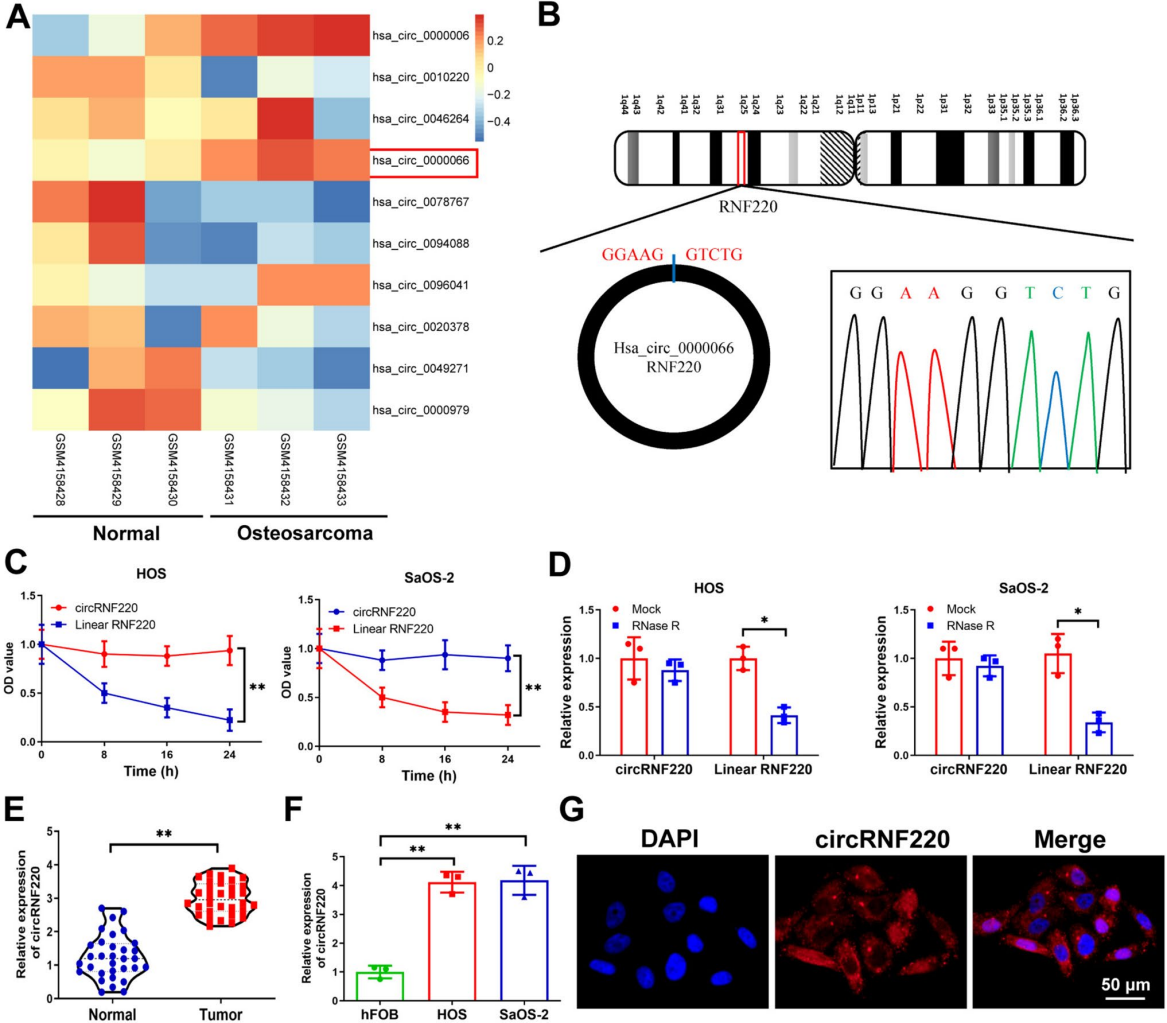
CircRNF220 was highly expressed in OS cells and tissues. **A** The differential expression of circRNAs from the GSE94591 database. **B** Sanger sequencing of the back splice junction of circRNF220. **C**, **D** Following RNase R or Actinomycin D treatment, the qRT-PCR graph shows circRNF220 and RNF220 expressions. **E**, **F** CircRNF220 expression in OS tissue and cells (G) Subcellular distribution of circRNF220 by FISH assay

In contrast to healthy controls, circRNA array analysis in GSE140256 indicated a high OS tissue level of circRNF220 (*P* = 0.0005, Fig. 1A). Sanger sequencing was then used to confirm the back-spliced region sequence (Fig. 1B). After the transcription inhibition by Actinomycin D, the half-life of circRNF220 was longer than that of linear RNF220 in HOS (*P* = 0.0065) and SaOS-2 (*P* = 0.0073) cells (Fig. 1C). Furthermore, circRNF220 was more RNase R resistant than RNF220 (Fig. 1D). The qRT-PCR data confirmed elevated circRNF220 levels in the OS cells and tissue (Fig. 1E, F). Moreover, FISH assays revealed that circRNF220 was predominantly localized in the cytoplasm (Fig. 1G). In addition, clinicopathological information demonstrated that high expression of circRNF220 was associated with TNM stage and tumor size (Supplementary Table 2). Overall, the results showed that circRNF220 is stable, and its expression gets upregulated in OS.

### Silencing circRNF220 inhibited OS progression

**Fig. 2:**
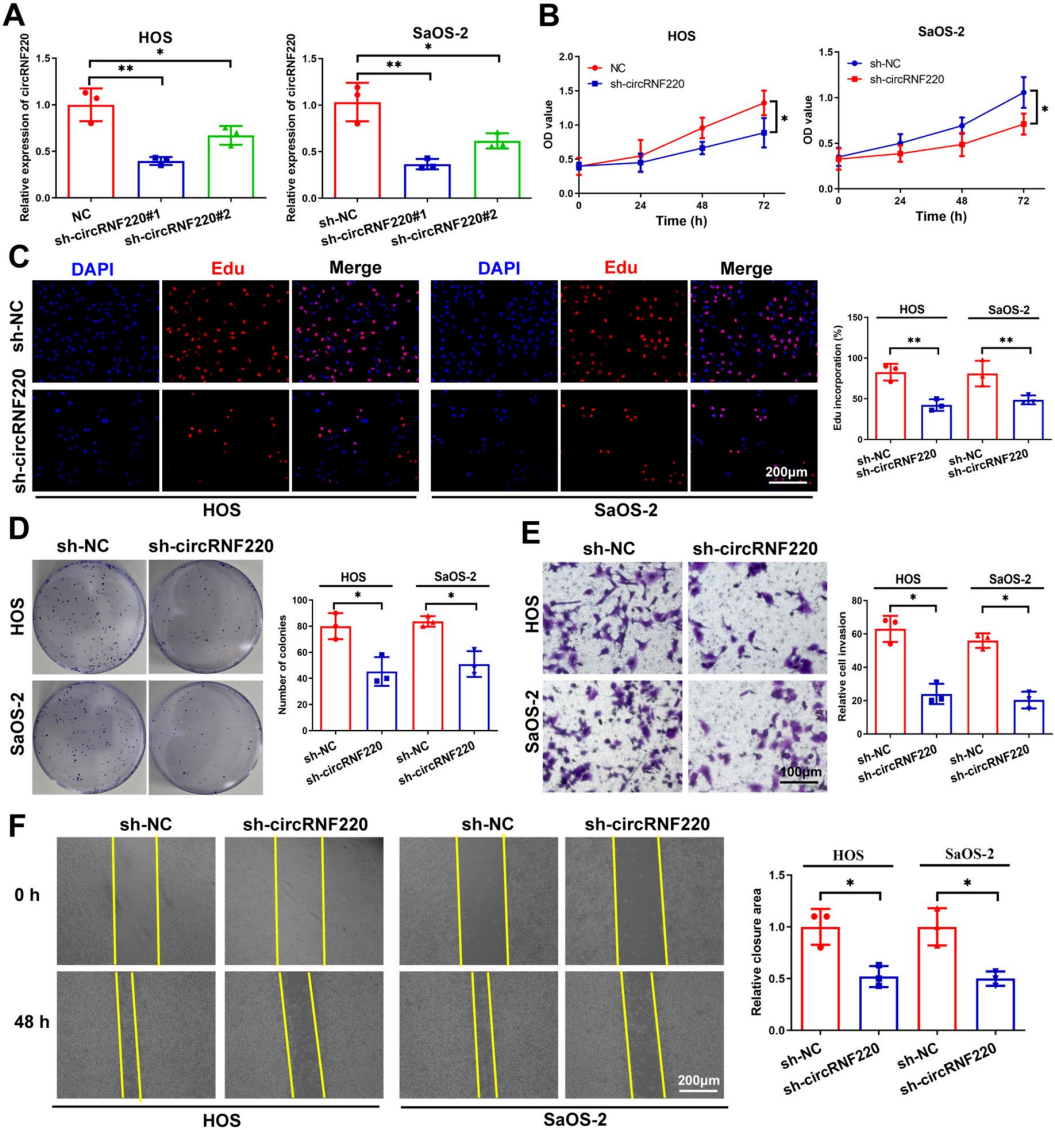
Silencing circRNF220 inhibited OS progression. **A** After transfection, the qRT-PCR graph showing circRNF220 and RNF220 expressions. **B**, **C** CCK-8 and EdU assay results showing cellular proliferative potential. **D** Colony formation assay in transfected OS cells (*n* = 3). **E**, **F** Transwell assay and wound-healing results show the cellular migratory and invasive potentials

The qRT-PCR analysis showed that treating cells with sh-circRNF220 led to the knockdown of circRNF220 (Fig. 2A). Since sh-circRNF220#1 had higher knockdown efficiency, it was used for further experiments. CCK-8 and EdU assays demonstrated that circRNF220 silencing led to the suppression of OS cell multiplication (Fig. 2B, C). Further, upon the circRNF220 knockdown, OS cells’ capacity to form colonies was suppressed (Fig. 2D). sh-circRNF220 also decreased the invasive and migratory abilities of OS cells in wound-healing and Transwell assays (Fig. 2E, F). These results collectively confirmed that silencing circRNF220 repressed OS progression in vitro.

### METTL3 enhanced circRNF220 expression via m6A modification

**Fig. 3:**
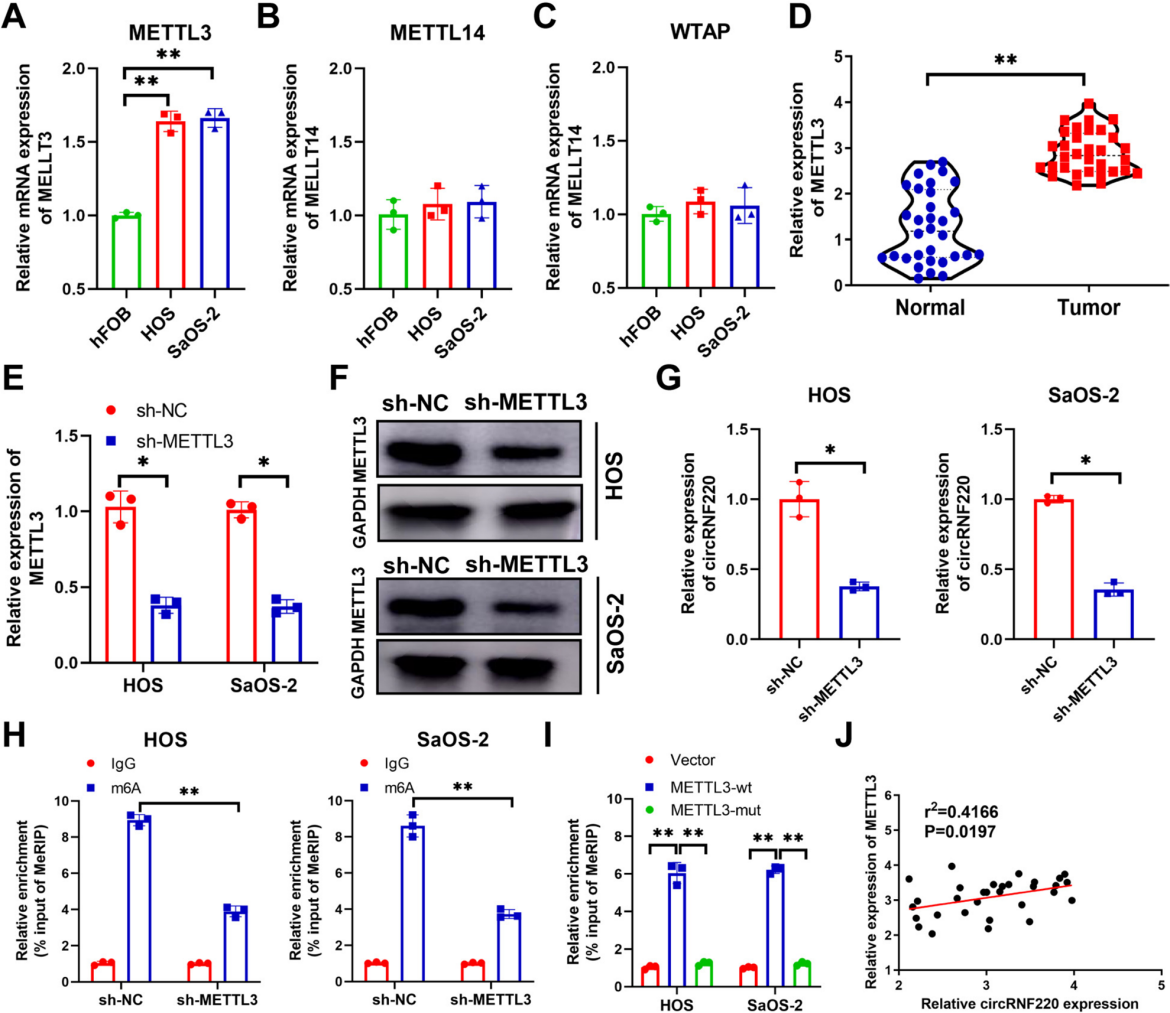
3.3 METTL3 enhanced circRNF220 expression via m6A modification. **A**, **B**, **C** The expression of METTL3, METTL14 and WTAP in hFOB and OS cells. **D** The expression of MELLT3 in OS tissues. **E**, **F** The mRNA and protein expressions of MELLT3. **G** The expression of circRNF220 in OS cells. **H** The m6A level of circRNF220 after knockdown of METTL3 in OS cells. **I** The m6A level of circRNF220 after transfection. **J** The relationship between METTL3 and circRNF220 expression

The expression of m6A writer enzymes (METTL3, METTL14, and WTAP) was detected using qRT-PCR. The results indicated that only METTL3 exhibited upregulation in OS cells compared to hFOB cells (Fig. 3A−C). Therefore, METTL3 was chosen for further investigation. In addition, the results revealed a significantly elevated expression of METTL3 in OS tissue samples (Fig. 3D). To investigate the potential mechanism by which METTL3 regulated circRNF220, we transfected sh-METTL3 to silence the mRNA and protein expression of METTL3 (Fig. 3E, F). The qRT-PCR results demonstrated a decrease in the expression of circRNF220 following the knockout of METTL3 (Fig. 3G). Furthermore, MeRIP-PCR detection revealed a significant reduction in the m6A level of circRNF220 after shMETTL3 transfection (Fig. 3H). Moreover, the m6A level of circRNF220 was increased by transfection with METTL3-wild type (wt). However, transfection with METTL3-mutant (mut) did not have any effect on the m6A level of circRNF220 (Fig. 3I). In addition, the expression of METTL3 was positively correlated to circRNF220 expression (Fig. 3J). These results revealed that METTL3 enhanced circRNF220 expression via m6A modification.

### CircRNF220 directly targeted miR-330-5p

**Fig. 4:**
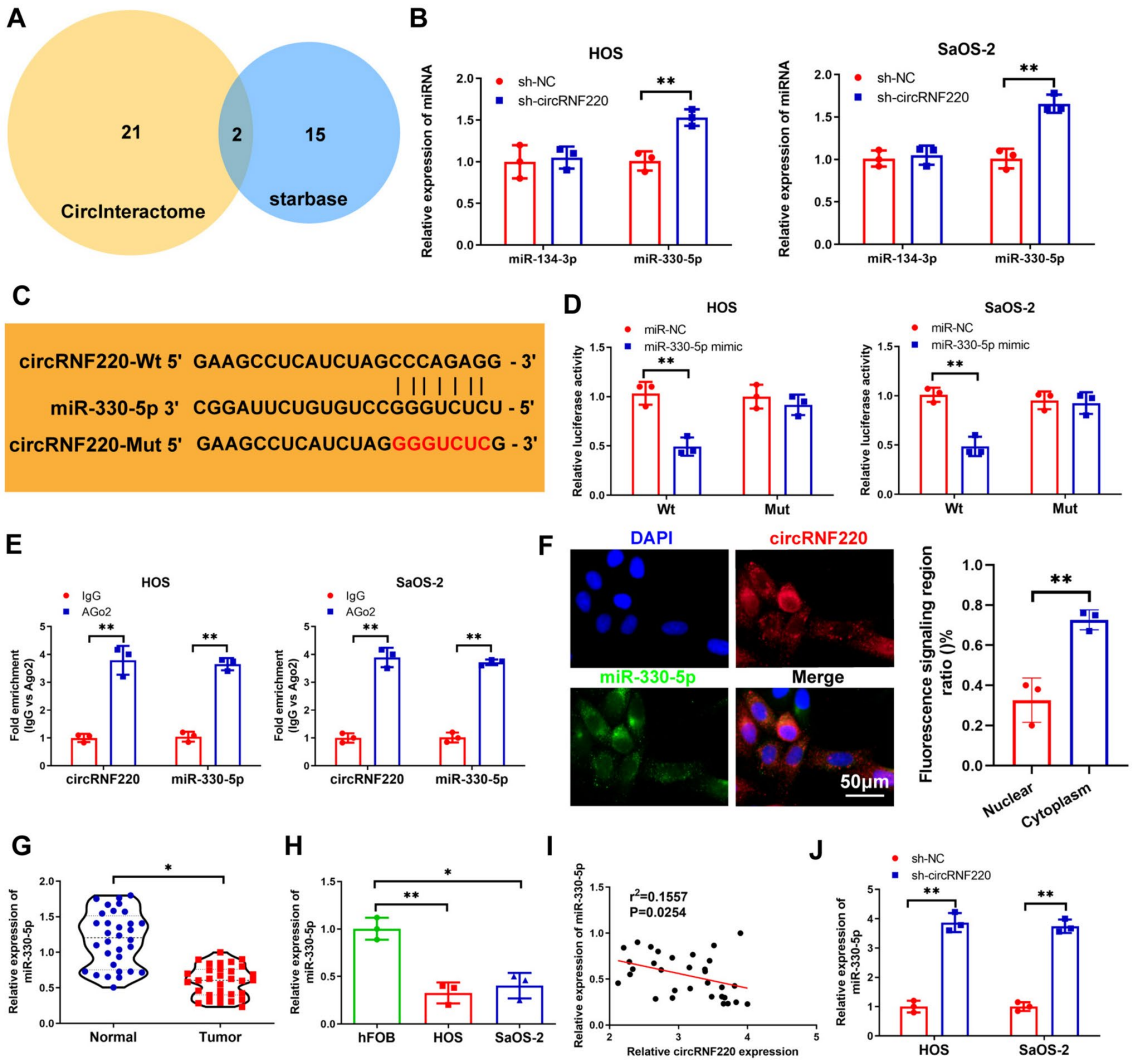
CircRNF220 directly targeted miR-330-5p. **A** The potential miRNAs based on overlapping predicted results. **B** The expression of miR-330-5p and miR-134-3p in OS cells after knockdown of circRNF220. **C** CircRNF220–miR-330-5p binding sites. **D** The relationship between miR-330-5p and circRNF220 identified by a Luciferase reporter assay. **E** RIP assay showing the interaction between CircRNF220 and miR-330-5p. **F** Cytoplasmic co-localization of circRNF220 and miR-330-5p shown by FISH assay. **G**, **H** The expression of miR-330-5p in OS tissues and cells was determined using qRT-PCR. **I** The negative relationship between circRNF220 and miR-330-5p. **J** The qRT-PCR outcome for sh-circRNF220’s role in the expression of miR-330-5p

Circinteractome and starbase, two online databases, were utilized to predict the target of circRNF220. The analysis revealed that two miRNAs potentially bound to circRNF220 based on overlapping predicted results (Fig. 4A). Subsequently, qRT-PCR results confirmed that silencing circRNF220 only led to increased expression of miR-330-5p in OS cells (Fig. 4B). Therefore, miR-330-5p was selected for further investigation. The results revealed a binding site for miR-330-5p in the circRNF220 sequence (Fig. 4C). Subsequently, overexpression of miR-330-5p had a suppressive effect on the relative luciferase activity of the circRNF220-wt reporter but had no effect on the circRNF220-mut reporter (Fig. 4D). The circRNF220–miR-330-5p interaction was further confirmed using RIP assay (Fig. 4E). Furthermore, the FISH assay revealed the cytoplasmic enrichment of circRNF220 and miR-330-5p (Fig. 4F). MiR-330-5p was shown to be downregulated in OS cells and tissues as compared to healthy tissues and cells (Fig. 4G, H). According to Pearson’s correlation outcomes, circRNF220 was negatively correlated to miR-330–5p in the OS tissues (Fig. 4I). Furthermore, miR-330–5p levels were upregulated in OS cells upon silencing of circRNF220 (Fig. 4J). Thus, circRNF220 directly targets miR-330-5p.

### MiR-330-5p inhibitor reversed the impairment of sh-circRNF220 on OS development

**Fig. 5:**
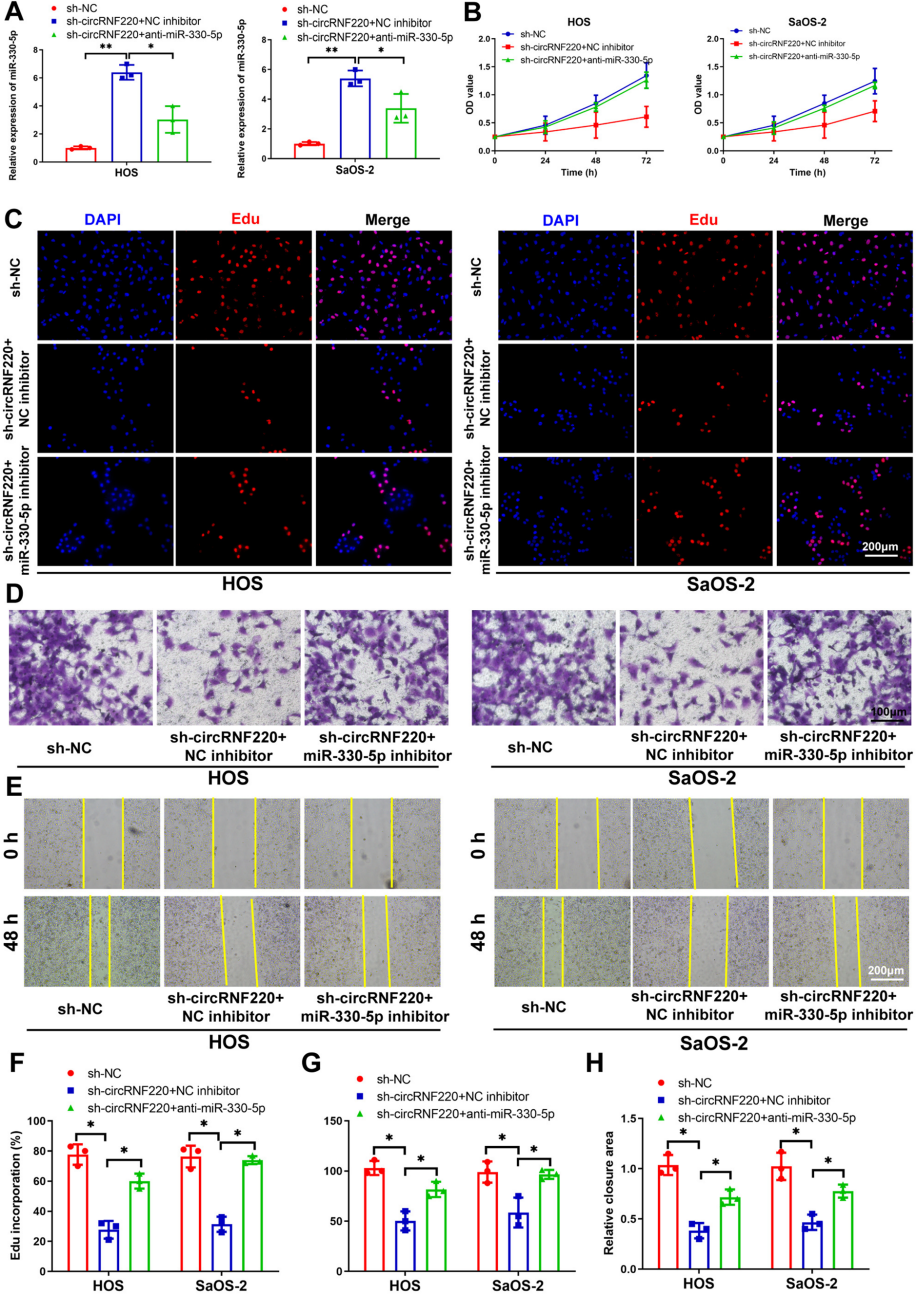
MiR-330-5p inhibitor reversed the impairment of sh-circRNF220 on OS development. **A** After co-transfection, the qRT-PCR-based analysis on the miR-330-5p level. **B**, **C**, **F** CCK-8 and EdU assays showing the proliferative potential of cells. **D**, **E**, **G**, **H** Wound-healing and Transwell assays reveal cellular migratory and invasive potentials

The qRT-PCR results showed that the miR-330-5p inhibitor decreases the sh-circRNF220-induced upregulation of miR-330-5p (Fig. 5A). Consequently, the sh-circRNF220-mediated suppression of cell proliferation, invasion repression, and migratory retardation were all counterbalanced (Fig. 5B–H). The above findings confirmed that circRNF220 regulates OS progression through miR-330-5p sponging.

### Survivin is a direct target of miR-330-5p.

**Fig. 6:**
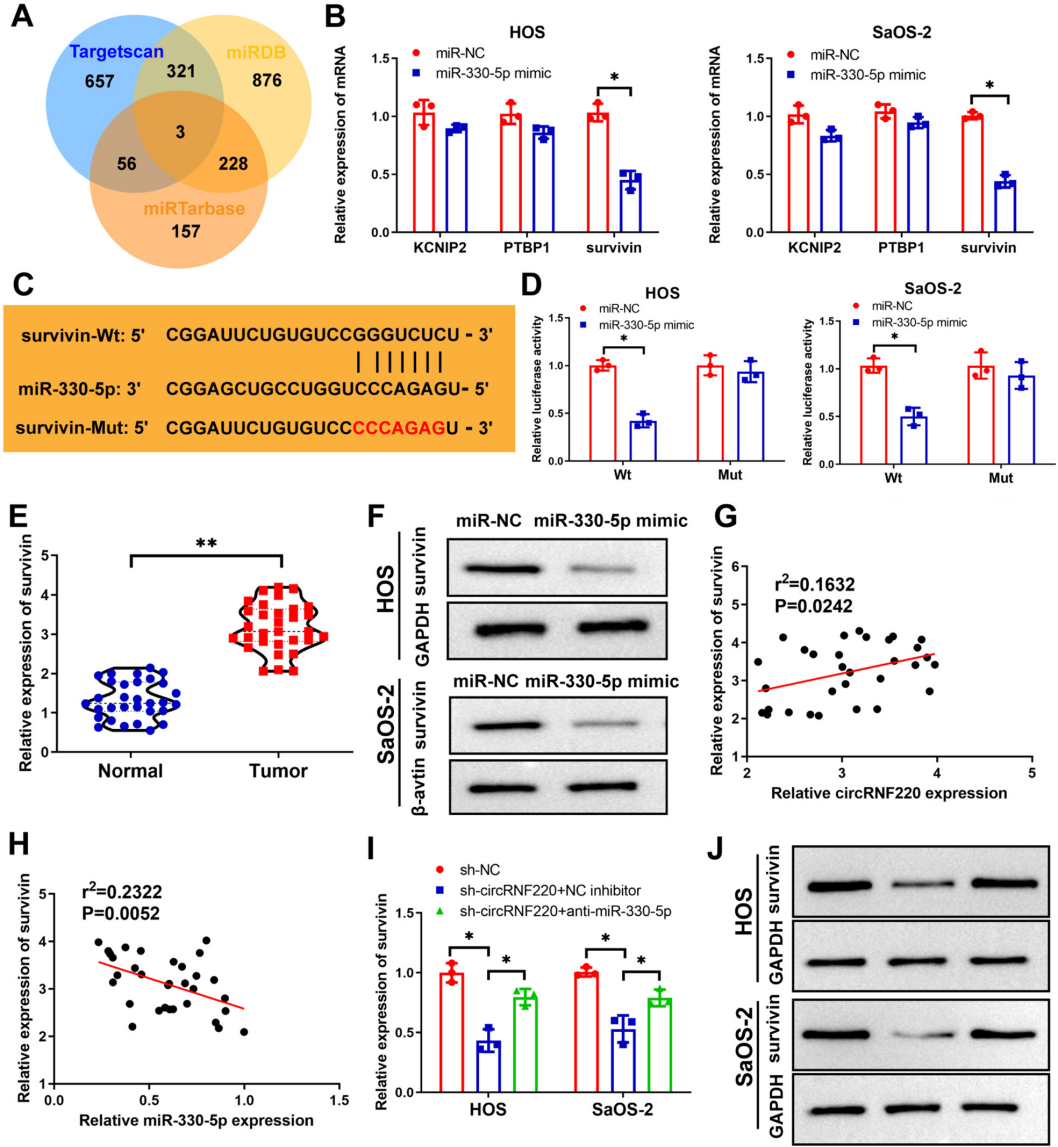
Survivin was a direct target of miR-330-5p. **A** The possible targets of miR-330-5p by bioinformatics analysis. **B** The expressions of potential genes binding to miR-330-5p. **C** Survivin’s binding sites interact with miR-330-5p. **D** Validation of miR-330-5p–survivin binding sites by the luciferase reporter assay. **E** A high OS tissue level of survivin is shown. **F** Western blot for protein expression of survivin. **G** Survivin was found to be linked negatively to miR-330-5p. **H** circRNF220 was positively related to survivin (**I**, **J**). The mRNA (**I**) and protein (**J**) expressions of survivin were assessed after co-transfection (*n* = 3)

Bioinformatics analysis was used to forecast potential targets (survivin, KCNIP2, and PTBP1) for miR-330-5p (Fig. 6A). The expression of survivin was significantly reduced following the miR-330-5p mimic transfection in OS cells (Fig. 6B). Survivin and miR-330-5p were shown to share binding sites (Fig. 6C). The Luciferase reporter assay showed that compared to the survivin 3’UTR-mut reporter, the survivin 3’UTR-wt reporter exhibited upregulated luciferase inhibition in the OS cells following miR-330-5p transfection (Fig. 6D). In addition, survivin was upregulated in OS tissues (Fig. 6E). Western blotting unraveled that the survivin protein levels were lowered by miR-330-5p mimic (Fig. 6F), and survivin is negatively correlated to the expression of miR-330-5p while positively correlated to circRNF220 expression (Fig. 6G, H). In addition, the knockdown of circRNF220 evoked mRNA and protein downregulation of survivin, while miR-330-5p inhibitor antagonized this inhibition (Fig. 6I, J). These findings demonstrated that circRNF220 upregulated the level of survivin through miR-330-5p sponging.

### CircRNF220 regulated OS progression by regulating the miR-330-5p/survivin axis

**Fig. 7:**
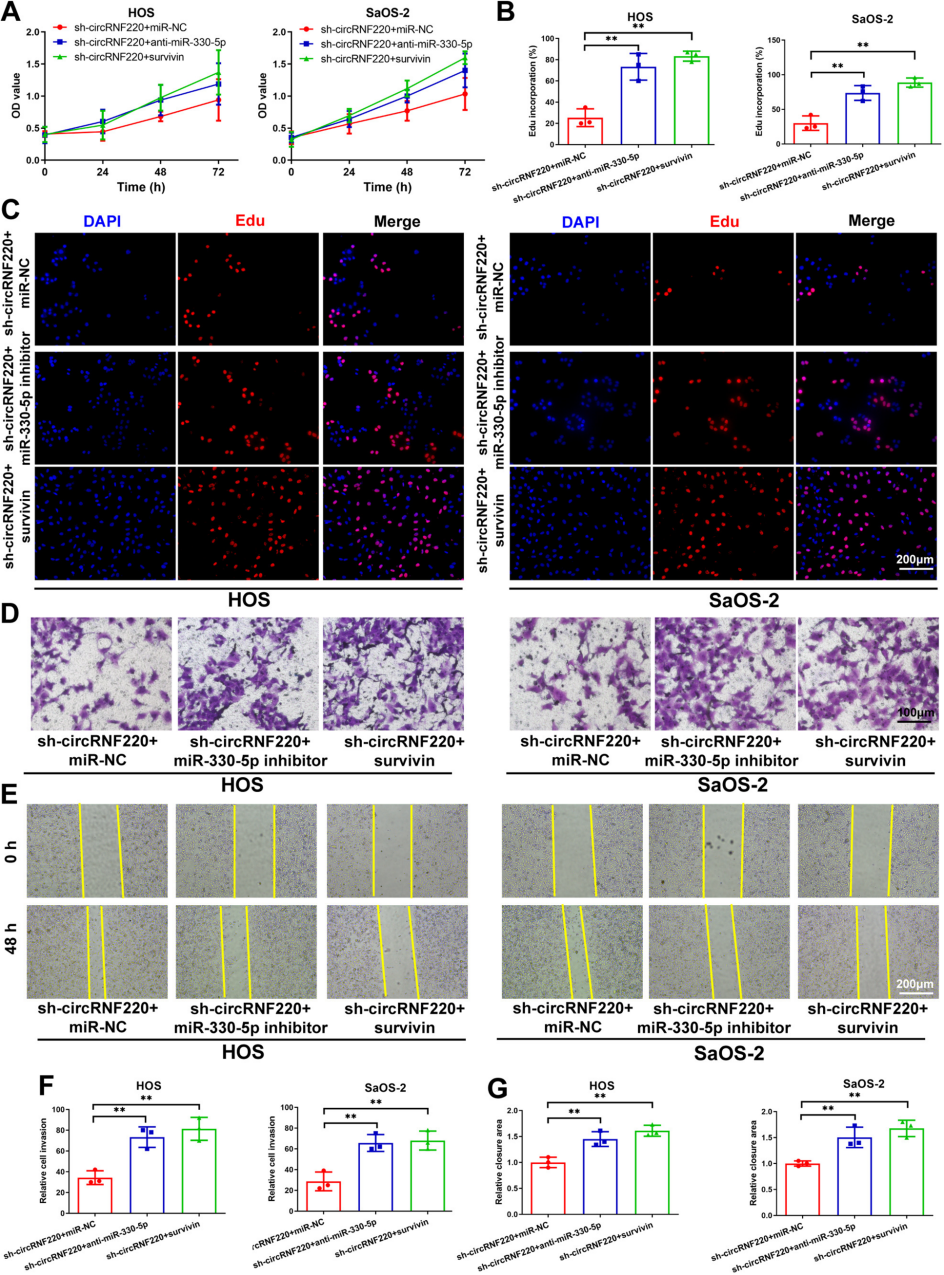
CircRNF220 regulated OS progression by regulating the miR-330-5p/survivin axis. **A**, **B**, **C** The determination of cell proliferation was performed by CCK-8 combined with EdU assays. **D**, **E**, **F**, **G** Transwell assays and wound-healing showing migratory and invasive potentials of OS cells

Survivin overexpression and sh-circRNF220 vectors were transfected into OS cells to examine whether circRNF220 facilitated OS progression through the miR-330-5p/survivin axis. The results showed that survivin overexpression and miR-330-5p inhibition partially reversed the circRNF220 knockdown-induced suppression of cellular multiplication, migration, and invasion (Fig. 7A–G).

### Down-regulation of circRNF220 inhibited the OS growth in vivo

**Fig. 8:**
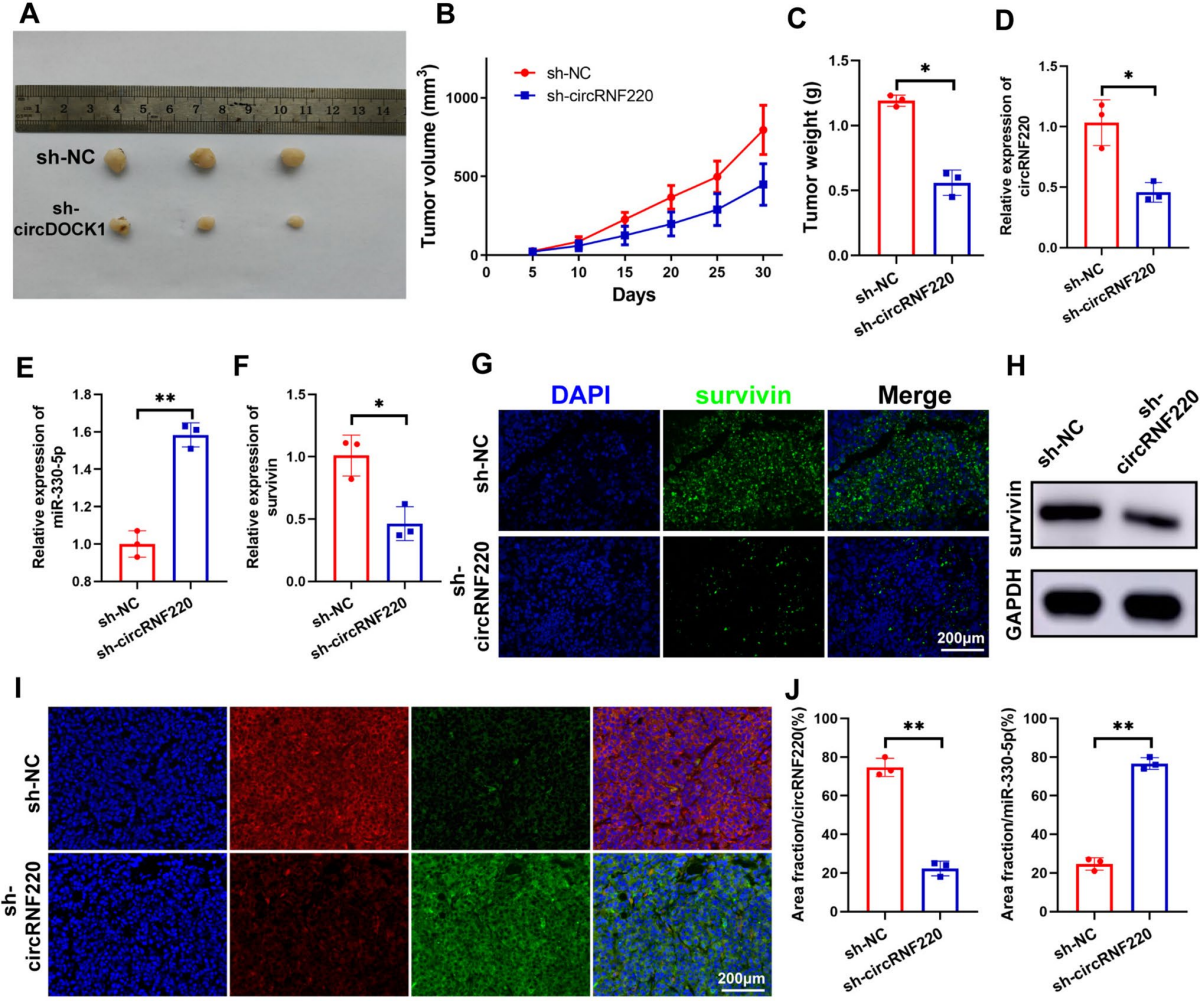
Down-regulation of circRNF220 inhibited OS growth in vivo. **A**, **B**, **C** After the knockdown of circRNF220, the tumor weight and volume are shown **D**, **E**, **F** OS tissue levels of circRNF220, miR-330-5p, and survivin. **G**, **H** Immunofluorescence staining and western blot was performed to evaluate the expression of survivin. **I** The co-location of circRNF220 and miR-330-5p in tumor samples. **J** The quantification if circRNF220 and miR-330-5p

The in vivo effect of circRNF220 was investigated using a xenografted model. In the sh-circRNF220 group, the volume and weight of tumors were reduced compared to the control (Fig. 8A–C). In addition, the expression of circRNF220 and survivin were decreased, while miR-330-5p expression was increased (Fig. 8D–F). Similarly, immunofluorescence and western blot assays showed that the survivin protein level was lower in the sh-circRNF220 group (Fig. 8G). In addition, FISH revealed the colocation between circRNF220 and miR-330-5p (Fig. 8H, I).

## Discussion

As a frequent juvenile primary osseous malignancy, the pathogenesis of OS has been extensively researched (Zhang et al. 2022b). The role of innumerable non-coding RNAs (ncRNAs) in OS regulation has been identified alongside the development of RNA sequencing (Celik et al. 2022; Liu and Shang 2022). The majority of non-coding RNA research has focused on circRNAs, some of which have been found to be involved in the carcinogenesis of OS, making them a possible diagnostic and therapeutic marker for the disease (Zhu et al. 2022; Huang et al. 2022; Yang et al. 2021). Circ_0000285, for example, is upregulated in OS, promoting malignant behavior via the miR-409-3p/IGFBP3 pathway (Long et al. 2020). According to previous studies, circRNF220 increases cancer cell multiplication, invasion and motility, exhibiting an oncogenic effect on cancer development (Zhang et al. 2022a; Liu et al. 2021). In this study, we have successfully demonstrated elevated levels of circRNF220 in both OS tissues and cells. Additionally, we have shown that inhibiting circRNF220 expression effectively suppresses the progression of OS. These findings strongly indicated that circRNF220 played a crucial role in promoting the malignant behaviors of OS at the cellular level.

The growing body of evidence suggests that m6A plays a significant role in human cancer, which has captured the interest of researchers (Ma et al. 2019). The modifications of the m6A gene are widespread and have been linked to the development of human cancer (Shen et al. 2021). For instance, Zhang et al. found that inhibiting m6A modification leads to a malignant phenotype in gastric cancer cells (Zhang et al. 2019). Previous studies have reported the involvement of METTL3, a catalytic subunit, in m6A modification. Specifically, it has been demonstrated that METTL3 promotes tumorigenesis in OS through an m6A-dependent mechanism (Wang et al. 2020d). However, no studies have demonstrated the regulatory role of METTL3 in circRNF220 in OS. In this research, we found that the expression of circRNF220 was enhanced through METTL3-mediated m6A modification.

CircRNAs compete with miRNAs as endogenous RNAs and then regulate the target gene expression (Man et al. 2021). For instance, circOMA1 regulates c-Myc expression through miR-1294 and promotes the progression of OS (Shi et al. 2022). Previous studies have shown the function of miR-330-5p in the tumorigenesis and progression of OS (Wang et al. 2019). In this study, RIP and dual luciferase assays validated the binding relationship between miR-330-5p and circRNF220. Importantly, silencing circRNF220 inhibited the proliferation, motility, and invasion of OS cells. However, when the miR-330-5p inhibitor was used, this effect was reversed, indicating that circRNF220 may act as a sponge for miR-330-5p, thereby promoting the malignant behaviors of OS cells.

Previous research has demonstrated that miRNAs can regulate gene expression at the post-transcriptional level by binding to the 3′-UTR of target genes (Tang et al. 2022). This regulatory mechanism enables them to influence the occurrence and progression of relevant diseases. In our current study, we conducted a luciferase assay to validate the interaction between miR-330-5p and survivin. Survivin, a member of the inhibitors of apoptosis (IAP) family, is known to be associated with the pathological phase, tumor infiltration, and metastasis (Erlandsson et al. 2022; Dong et al. 2022). Our findings indicated a significant reduction in survivin protein levels following the overexpression of miR-330-5p, suggesting that miR-330-5p may downregulate survivin by directly binding to it.

Several studies have shown that circRNAs regulate target mRNA expression by adsorbing certain miRNAs as competing RNAs (Hu et al. 2021). Our study demonstrated that circRNF220 played a role in promoting malignancy in OS cells by acting as a sponge for miR-330-5p. Based on the ability of miR-330-5p to bind to survivin, we proposed that circRNF220 mediated the miR-330-5p/survivin pathway to facilitate malignancy in OS cells. Through various functional assays, we found that silencing circRNF220 led to a decrease in cellular multiplication, motility, and invasion. However, these effects were reversed when survivin was overexpressed or miR-330-5p was suppressed, suggesting that circRNF220 regulated the progression of OS through the miR-330-5p/survivin pathway.

However, there are some limitations in our study. Survivin protein is one of the minimum anti-apoptotic proteins, and it plays a role in cellular stress response apoptosis and cell cycle (Renner et al. 2016). Previous studies have shown that survivin positive circulating tumor cells were associated with the prognosis of OS (Lu et al. 2023). However, the underlying role of survivin in OS remains unclear and requires further investigation.

## Conclusions

In conclusion, METTL3-mediated circRNF220 modulated the miR-330-5p/survivin axis to regulate the progression of OS. This study offers new insights into the pathogenesis of OS and proposes a potential therapeutic target for its treatment.

### Supplementary Information

Below is the link to the electronic supplementary material.

Supplementary file1 (DOCX 19 KB)

## Data Availability

The data are available from the corresponding author upon request.
