# Supplementary material for: METTL3-mediated m6A modification of circRNF220 modulates miR-330-5p/survivin axis to promote osteosarcoma progression
Source: J Cancer Res Clin Oncol. 2023 Oct 14;149(19):17347–60. doi: 10.1007/s00432-023-05455-x (PMC10657300; doi:10.1007/s00432-023-05455-x)
Supplement: Supplementary file 1 — Supplementary file1 (DOCX 19 KB) [file 432_2023_5455_MOESM1_ESM.docx]

| CircRNF220 | Forward  (divergent） | 5’- TGCTGTCTCTGGCCTCATTTC -3’ |
| --- | --- | --- |
|  | Reverse  (divergent) | 5’- GGGAATCATTCCCTCCTAAGAC -3’ |
| CircRNF220 | Forward  (convergent） | 5’- CCTTTCACCAACGGTTCCTA -3’ |
|  | Reverse  (convergent) | 5’- ATCTTCTTCCCAGTGTCATCAA -3’ |
| miR-330-5p | Forward | 5’- GCGTCTCTGGGCCTGTGTC -3’ |
|  | Reverse | 5’- AGTGCAGGGTCCGAGGTATT -3’ |
| survivin | Forward | 5’- GGACCACCGCATCTCTACAT -3’ |
|  | Reverse | 5’- GCACTTTCTTCGCAGTTTCC -3’ |
| GAPDH | Forward | 5’- GCTGAACGGGAAGCTCACTG -3’ |
|  | Reverse | 5’- GTGCTCAGTGTAGCCCAGGA -3’ |
| U6 | Forward | 5’- CGCTTCGGCAGCACATATAC-3’ |
|  | Reverse | 5’-TTCACGAATTTGCGTGTCAT-3’ |

**Supplementary table 1. Primer sequences in this study**

**Supplementary Table 2: The correlation between clinicopathological characteristics of OS patients and circRNF220 expression.**

| **Clinicopathological Characteristics** | **Number of Patients (n = 32)** | **Expression of circRNF220** | | ***P-*value** |  |
| --- | --- | --- | --- | --- | --- |
|  |  | **Low (n = 15)** | **High (n = 17)** |  |  |
| Gender |  |  |  | 0.7235 |  |
| male | 15 | 8 | 7 |  |  |
| female | 17 | 7 | 10 |  |  |
| Age (years) |  |  |  | 0.7345 |  |
| <18 | 18 | 9 | 9 |  |  |
| ­­≥18 | 14 | 6 | 8 |  |  |
| Tumor size (cm) |  |  |  | **0.0269** |  |
| <5 | 12 | 9 | 3 |  |  |
| ≥5 | 6 | 14 |  |  |  |
| TNM stage |  |  |  | **0.0105** |  |
| I-IIA | 19 | 5 | 14 |  |  |
| IIB-III | 13 | 10 | 3 |  |  |
| Anatomic location |  |  |  | 0.2907 |  |
| Tibia/femur | 20 | 11 | 9 |  |  |
| Elsewhere | 12 | 4 | 8 |  |  |
